# Synthesis of cobalt- ferrite and zinc oxide metal nanoparticles based-bentonite using SDS and their investigation as catalysts in synthesis of benzylbarbiturocoumarins

**DOI:** 10.3389/fchem.2024.1434488

**Published:** 2024-08-12

**Authors:** Pegah Baminejhad, Enayatollah Sheikhhosseini, Mahdieh Yahyazadehfar

**Affiliations:** Department of Chemistry, Kerman Branch, Islamic Azad University, Kerman, Iran

**Keywords:** CoFe_2_O_4_@ZnO@Bentonite, bentonite, reusable catalyst, multi-component reaction, green synthesis, benzylbarbiturocoumarins

## Abstract

In this research, a suitable and efficient CoFe_2_O_4_@ZnO@Bentonite nano-catalyst was designed and synthesized by using zinc oxide (ZnO) and cobalt ferrite (CoFe_2_O_4_) nanoparticles and bentonite by microwave irradiation. Characteristics of the synthesized nanocomposite were investigated by Fourier transform infrared (FT-IR), scanning electron microscope (SEM), energy dispersive X-ray (EDX), transmission electron microscope (TEM), X-ray diffraction (XRD), Bruner- Emmett-Teller (BET) and vibrating sample magnetometer (VSM) techniques. The produced catalyst was effectively employed as a supported solid acid catalyst in mildly agitated three-component reactions involving aromatic aldehydes, 4-hydroxycoumarin, and 1,3-dimethyl-barbituric acid in a single pot to produce benzylbarbiturocoumarins. Starting materials were condensed via three C–C bond formation by CoFe_2_O_4_@ZnO@Bentonite as an efficient, recyclable, and environmentally safe nanocatalyst to obtain target products. The advantages of this method include using a natural substrate, small amounts of catalyst, aqueous media, performing reactions at ambient temperature, simple separation and purification of products, and good yields with short reaction times.

## 1 Introduction

Scientists and engineers from several scientific disciplines have become interested in nanoparticles (NPs) in recent years since they have become increasingly important in various sectors. Because of their distinct characteristics over bulk materials, including high surface area, high surface-to-volume ratio, tunable porosity, controllable particle size, and size-dependent characteristics like improved catalytic properties and biological applications, nanoparticles (NPs) are appealing for a variety of uses (Ahankar et al., 2019).

Doping is a popular method of modifying NPs’ surface characteristics to improve their antimicrobial characteristics, catalytic activity, etc. Furthermore, doping makes it possible to achieve desirable qualities for particular uses, including the removal of hazardous colors via adsorption and the treatment of wastewater and nuclear waste (Dubadi et al., 2023). Of all the NP subclasses, metal NPs (MNPs) and metal oxide NPs (MONPs) are very significant.

The process of creating novel, targeted catalysts and investigating their catalytic activity has had a significant impact on increasing the effectiveness of a variety of organic synthesis processes. By displacing nonselective, unstable, or hazardous catalysts, the development of such catalysts has led to more affordable and ecologically friendly chemistry. Lewis acid and Lewis base characteristics are present on the surface of metal oxides. This is a feature shared by several metal oxides, including TiO_2_, Al_2_O_3_, and ZnO, which are great adsorbents for a broad range of organic molecules and raise the reactant’s reactivity. Because of their surface characteristics, which imply the possibility of a highly rich organic chemistry, zinc oxide nanoparticles are among the most intriguing multifunctional metal oxides (Alinezhad and Salehian, 2013; Al-Naggar et al., 2023).

Nanomaterials can be metals, ceramics or polymers. Meanwhile, ceramic nanostructures are considered one of the most important and applied branches of nanomaterials due to their special properties. among nanoceramics, zinc oxide nanoparticles are certainly some of the most interesting multifunctional of metal oxide, because they have surface properties that suggest that a very rich organic chemistry may occur there (Kora, 2024).

Zinc oxide is an n-type semiconductor of the group (II-VI) with a 3.37 eV band at room temperature, which is mostly crystallized with Wurtzite structure and hexagonal symmetry. In comparison with other semiconductors with a wide band gap such as GaS and ZnSe, the excitation energy of ZnO is very high (60 MeV), which makes its excited state stable at room temperature (Sathitsuksanoh et al., 2010; Amrute et al., 2021).

In addition, zinc is a rare and essential element in bone tissues, skin, muscle, brain, and various enzyme systems (Alimohamady et al., 2019; Huang et al., 2021). Because of this, zinc oxide nanoparticles have been shown to have antibacterial and antifungal properties and are utilized as food and medication additives (Machotova et al., 2020; Ansari et al., 2011; He et al., 2011). Numerous nanostructures, such as nanoneedles, nanobelts, nanoflowers, nanorods, nanobows, nanonails, nanoparticles, and nanowires, have been seen in ZnO (Eftekhari et al., 2006).

In recent years, there has been a lot of fascination with chemical reactions to create magnetic composite nanoparticles, especially those of various ferrites, which are a green, non-toxic catalyst that can be easily isolated from aqueous solutions and reused.

The excellent catalytic activity of magnetic nanoparticles is influenced by their high surface area, compact size, remarkable chemical stability, strong electromagnetic characteristics, and active sites.

Cobalt ferrite is a strong magnetic material having the property of ferromagnetism. It is distinguished by an inverted spinel structure in which Fe^3+^ ions occupy all of the tetrahedral sites (A-sites) and half of the octahedral sites (B-sites). On the other hand, Co^2+^ ions are dispersed across the octahedral B-sites (Fallah et al., 2023).

The intriguing characteristics of cobalt ferrite nanoparticles include their strong magnetocrystalline anisotropy (a positive anisotropy constant), and therefore, good mechanical toughness, modest saturation magnetization, a high demagnetizing field at ambient temperature, and high chemical resistance, high coercivity, very high electrical resistance, electrical insulation and high magnetostriction have catalytic and functional properties such as being used in molecular imaging in the medical field, magnetic data storage (Yanez-Vilar et al., 2009), sensors, ferrofluids and biomedical applications, high-frequency magnets, information storage systems, bulk magnetic cores, high-density recording media, magnetic fluids (Zhao et al., 2007).

These applications of cobalt ferrite nanoparticles are due to its ability to distribute cations among sub-lattices in tetrahedral and octahedral sites. Different methods have been proposed for the synthesis of cobalt ferrite nanoparticles, including sol-gel method, chemical co-precipitation, spray co-precipitation, forced hydrolysis in a polyol system, synthesis in oil-in-water micelles, synthesis in reverse micelles, hydrothermal method, spray drying, solid-state, microemulsion processes, and mechanical alloying (Modabberasl et al., 2024).

Montmorillonite is an aqueous aluminosilicate containing a small amount of alkali and alkaline earth metal ions. The unit cell of montmorillonite is two silicon-oxygen tetrahedral layers with a hydrogen-oxygen octahedral layer. The cation heterovalent *homoimage* substitution makes it charge and has various characteristics such as adsorption and cation exchange. Bentonite, also known as bentonite, bentonite, is a kind of clay or clay rock with montmorillonite as the main mineral component (Otsuki and Hayagan, 2020).

Al_2_O_3_.4SiO.H_2_O is the chemical formula of bentonite, a water-based clay mineral montmorillonite that exhibits several unique properties, including substantial external surface area, excellent adherence, and strong dispersibility in organic and aqueous solutions (Nagy et al., 2004).

Clays are naturally occurring aluminosilicates having a sheet’s structure. Bentonite swells in water and it readily absorbs a wide range of compounds. Due to this reason, it is used as a catalyst and catalyst support in the chemical processes (Venkatathri, 2006). Bentonite is used in the synthesis of absorbents, and pharmaceutical products, and in the medical field as a base for many skin formulations (Ravindra Reddy et al., 2017).

Bentonite functions as a binder to hold together mineral particles in an aqueous solution. The electrostatic interaction between mineral particles and bentonite can explain this phenomenon (Otsuki and Hayagan, 2020).

Bentonite was employed in this work as the primary adsorbent for the nanoparticles. Due to its huge surface area, high adsorption capacity, cheap cost, and great swelling capability, it is advantageous for the treatment of environmental contamination (Nagy et al., 2004; Venkatathri, 2006).

Bentonite has been used as a binder of oxide material like; iron, zinc, chromium, Cu, cobalt, and titanium oxides for SO_2_ dibenzothiophene, and Chattonella marina (Otsuki and Hayagan, 2020; Hatvani-Nagy et al., 2024).

Recently, the primary goal of organic synthesis has been to create structurally complex organic molecules from simple substrates.

**FIGURE 1:**
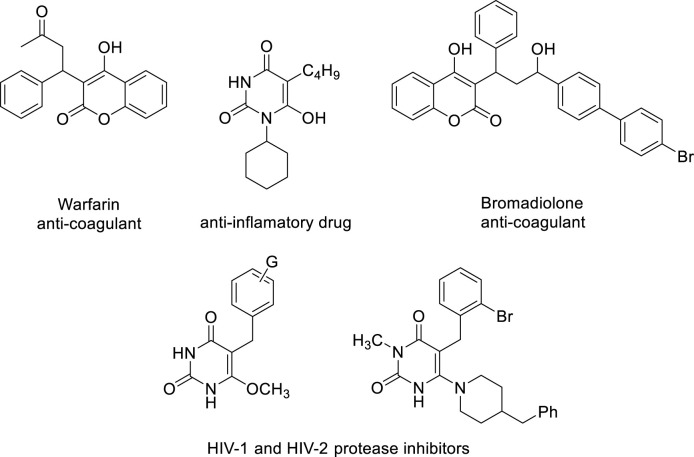
Samples of biologically active compounds based on 4-hydroxycoumarin and barbituric acid structures.

One of the significant heterocyclic compounds that chemists and pharmaceuticals are still interested in is barbituric acid (Mahmudov et al., 2014). Based on this molecule, several multicomponent processes have been developed for the production of new heterocycles (Kazemi-Rad et al., 2014; Siddiqui, 2015). It may trigger an array of impacts, from mild sedation to complete anesthesia (Mahmudov et al., 2014; Baruah et al., 2012), in addition to negative reactions like hypnotics, anxiolytics, and anticonvulsants (Mahmudov et al., 2014; Baruah et al., 2012), and also has to decrease fat deposition in non-alcoholic fatty liver disorder (Ma et al., 2011), and anti-cancer activity (Dhorajiya et al., 2014), due to a variety of biological and pharmacological characteristics, such as its central nervous system depressant properties. Additionally, this material and its derivatives have demonstrated the ability to sequester metals (Singh et al., 2010) and were employed as a new anchor unit for dye-sensitized solar cells (Irgashev et al., 2014). Combining barbituric acid with extra pharmacophoric groups and the 4-hydroxycoumarin moiety may produce new substances with potential biological action. For example, scaffold 4-hydroxycoumarin derivatives are present in a wide range of pharmaceutical and biological substances, including bromadiolone, warfarin, coumatetralyl, phenprocoumon, and carbochromen (Ghosh and Das, 2012; Talhi et al., 2015), among many others. Anticoagulant (Jung et al., 2001), antioxidant (Melagraki et al., 2009), anti-HIV (Hesse and Kirsch, 2002), Antibacterial (Ghosh and Das, 2012), antiviral (Lee et al., 1998), and qualities have also been shown for these compounds. Many physiologically active chemicals and drugs include barbituric acid or 4-hydroxycoumarin fragments (Figure 1).

Several approaches used to create benzylbarbiturocoumarins, such as graphene oxide (GO) (Eskandari and Karami, 2016) and silica sodium carbonate (SSC) (Eskandari et al., 2016), have proven beneficial and efficient; yet, the techniques also have their shortcomings, such as the use of tainted organic solvent (EtOH) and lengthy reaction times.

In continuation of our attempts to design and extension of novel catalytic systems and their application in the preparation of heterocyclic compounds via MCR methodologies, this work, reported that synthesized environment-friendly CoFe_2_O_4_@ZnO@Bentonite catalyst led to the preparation of benzyl-barbituric coumarins from the condensation reaction of 4-hydroxycoumarin, a wide range of aryl aldehydes and barbituric acid/N,N-dimethylbarbituric acid by the new methodology.

## 2 Experimental section

### 2.1 Chemicals and reagents

Cobalt acetate (MW = 177.02 g/mol), iron nitrate (MW = 404 g/mol), sodium dodecyl sulfate (SDS) (MW = 288.37 g/mol), ammonia (MW = 17.03 g/mol), sodium hydroxide (MW = 40 g/mol), zinc acetate (MW = 183.48 g/mol), oxalic acid (MW = 90.03 g/mol) and bentonite (MW = 224.145 g/mol) were also obtained from Merck company. All the organic solvents used for the washing purpose were directly used without further purification. Double-distilled water was used for the solution preparation.

### 2.2 Material characterization

The crystallinity, phase structure, and crystallite size of CoFe_2_O_4_@ZnO@Bentonite nanocatalyst was determined through a PC-APD X-ray diffractometer and Kα radiation (α_2_, λ_2_ = 1.54439 Å) and graphite mono-chromatic Cu radiation (α_1_, λ_1_ = 1.54056 Å) (Philips, the Netherlands). The X’Pert HighScore Plus software was used for data analysis. The XRD pattern was obtained in the range 2°–70° 2θ, with a step of 0.016°. Then, scanning electron microscopy (SEM) and energy dispersive spectrometer (EDS) (KYKY & EM 3200) have been applied to observe CoFe_2_O_4_@ZnO@Bentonite nanocatalyst. Thermal behavior analysis was done in N_2_ between room temperature and 800°C using a STA-1500 thermoanalyzer. N_2_ adsorption–desorption isotherms (BET) were measured on a TriStar II Plus surface area and porosity analyzer at 77 K. Magnetization measurements were carried out with a Lakeshore (model 7407) under magnetic fields at room temperature. Thin layer chromatography (TLC) was applied to determine the purity of the substrates and reaction progress. An Electrothermal 9100 was used to determine the melting points, while FT-IR spectroscopy was attained on a Perkin-Elmer 240-C. Records of NMR spectra were obtained on a Bruker Avance spectrometer at 400 MHz for ^1^H NMR and 100 MHz for ^13^C NMR in CDCl_3_ and DMSO-*d*_*6*_ as the solvent, while tetramethylsilane (TMS) was considered as the internal reference.

### 2.3 Preparation of nanoparticles

#### 2.3.1 Preparation of cobalt ferrite nanoparticles

Cobalt acetate (Co(C_2_H_3_O_2_)_2_, 2.824 mmol, 0.5 g) and sodium dodecyl sulfate (C_12_H_25_NaSO_4_, 0.867 mmol, 0.25 g) were added to an aqueous solution of iron nitrate (Fe (NO_3_)_3_ · 9 H_2_O, 4.010 mmol, 1.62 g) followed by dropwise addition of sodium hydroxide (NaOH, 1 M) to enhance the pH of the solution to 10. After 1 day of stirring and completion of the reaction, the brownish precipitates were washed with deionized water and ethanol in a centrifuge, dried in an oven at 80°C, and calcined in a furnace at 800^°^C for 3 h (Nabiyouni et al., 2014).

#### 2.3.2 Synthesis of zinc oxide nanoparticles

Zinc acetate (16.350 mmol, 3 g) and oxalic acid (22.215 mmol, 2 g) were ground in a mortar for 1 h followed by 1 h of calcination at 450^°^C (Alinezhad and Salehian, 2013).

#### 2.3.3 Synthesis of CoFe_2_O_4_@ZnO@Bentonite nanocatalyst

**SCHEME 1:**
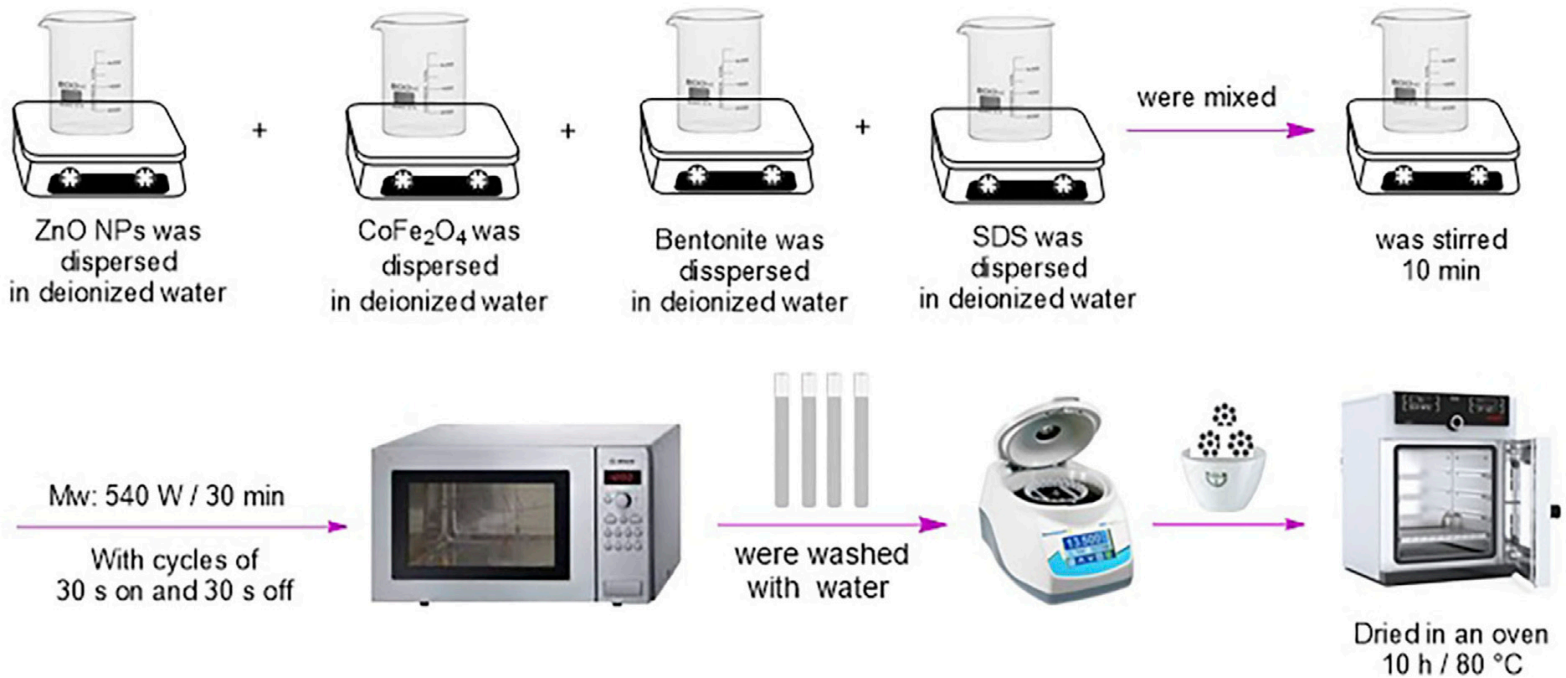
Synthesis of CoFe_2_O_4_@ZnO@Bentonite nanocatalyst.

Cobalt ferrite (0.5 mmol, 0.117 g), zinc oxide (0.5 mmol, 0.041 g), bentonite (3 mmol, 0.841 g), and SDS (0.867 mmol, 0.25 g) (as the stabilizer) were individually transferred into four beakers and with a minimum amount of water, magnetically stirred for 25 min to be dispersed. Then they mixed and were ultrasonicated for half an hour at the power of 540 W. The obtained precipitates were dried in an oven at 80 ℃ for 8 h (Scheme 1).

### 2.4 General process of preparing benzylbarbiturocoumarin derivatives using CoFe_2_O_4_@ZnO@Bentonite nanocatalyst

In a 100 mL flask equipped with a magnetic stirrer, a mixture of 1,3-dimethylbarbituric acid (6 mmol), 4-hydroxycoumarin (6 mmol), and different aromatic aldehydes (6 mmol) in the presence of 20 wt.% nanocatalyst of CoFe_2_O_4_@ZnO@Bentonite were prepared under optimal of water solvent, amount of 20 wt.% and at ambient temperature. The catalyst was extracted from the product by filtering the precipitates, which ended up dissolving in a small quantity of dimethyl sulfoxide once the reaction was finished. The catalyst’s activity was monitored by TLC (EtOAc/n-hexane, 2:3). Then water was added to the filtered solution to form a pure precipitate of the product. In some cases, the products were washed with water or ethanol for further purification.

### 2.5 Selected spectral data

*6-hydroxy-5-*(*2-(4-hydroxy-2-oxo-2H-chromen-3-yl)-1-*(*3-nitrophenyl*)*ethyl*)*-1*,*3-dimethylpyrimidine-2*,*4(1H*,*3H)-dione* (*4d*): m.p.:193–194°C. Yield: 95%. IR (KBr, cm^-1^): 3,431, 3,073, 1705, 2,959, 1,651, 1,618, 1,573, 1,529, 1,494, 1,346, 1,190, 1,102, 761. ^1^H NMR (400 MHz, DMSO-*d*_*6*_, ppm): δ = 3.08 (s, 3H, CH_3_), 3.27 (s, 3H, CH_3_), 5.61 (s, 1H, CH), 7.54 (d, *J*_1_ = 4 Hz, 2H, H-Ar), 7.71 (d, *J*_1_ = 4 Hz, 2H, H-Ar), 7.81 (d, *J*_1_ = 8 Hz, 2H, H-Ar), 8.27 (d, *J*_1_ = 8 Hz, 1H, H-Ar), 8.73 (s, 1H, H-Ar), 12.52 (s, 1H, OH), 12.53 (s, 1H, OH). ^13^C NMR (100 Hz, DMSO-*d*_6_, ppm): δ = 28.2, 28.4, 36.2, 119.9, 120.1, 123.9, 124.0, 124.3, 129.8, 130.1, 130.3, 132.0, 132.3, 133.9, 134.0, 146.0, 146.1, 151.1, 153.6, 160.0, 161.2.

*5-*(*1-(5-bromo-2-hydroxyphenyl)-2-*(*4-hydroxy-2-oxo-2H-chromen-3-yl*)*ethyl*)*-6-hydroxy-1*,*3-dimethylpyrimidine-2*,*4(1H*,*3H)-dione* (*4g*): m.p.: 190–192°C. Yield: 97%. IR (KBr, cm^-1^): 3,473, 3,365, 2,967, 2,933, 1,675, 1,652, 1,603, 1,568, 1,425, 1,385, 818, 757. ^1^H NMR (400 MHz, DMSO-*d*_*6*_, ppm): δ = 3.14 (s, 3H, CH_3_), 3.31 (s, 3H, CH_3_), 5.85 (s, 1H, CH), 6.67 (d, *J*_1_ = 8.4 Hz, 1H, H-Ar), 7.13 (dd, *J*_1_ = 8.8 Hz, *J*_2_ = 2 Hz 1H, H-Ar), 7.17 (s, 1H, H-Ar), 7.32 (d, *J*_1_ = 6.8 Hz, 1H, H-Ar), 7.66 (d, *J* = 8.4 Hz, 1H, H-Ar), 7.74 (t, *J* = 8.8 Hz, 2H, H-Ar) ppm, 11.66 (s, 1H, OH), 12.38 (brs, 2H, OH); ^13^C NMR (100 Hz, DMSO-*d*_6_, ppm): δ = 28.5, 30.3, 39.4, 58.4, 92.0, 104.4, 109.4, 118.0, 123.4, 124.8, 125.3, 126.3, 127.7, 128.7, 129.6, 132.6, 134.7, 152.7, 154.1, 163.5, 164.8, 168.0, 172.1.

## 3 Results and discussion

### 3.1 Characterization of CoFe_2_O_4_@ZnO@Bentonite

**FIGURE 2:**
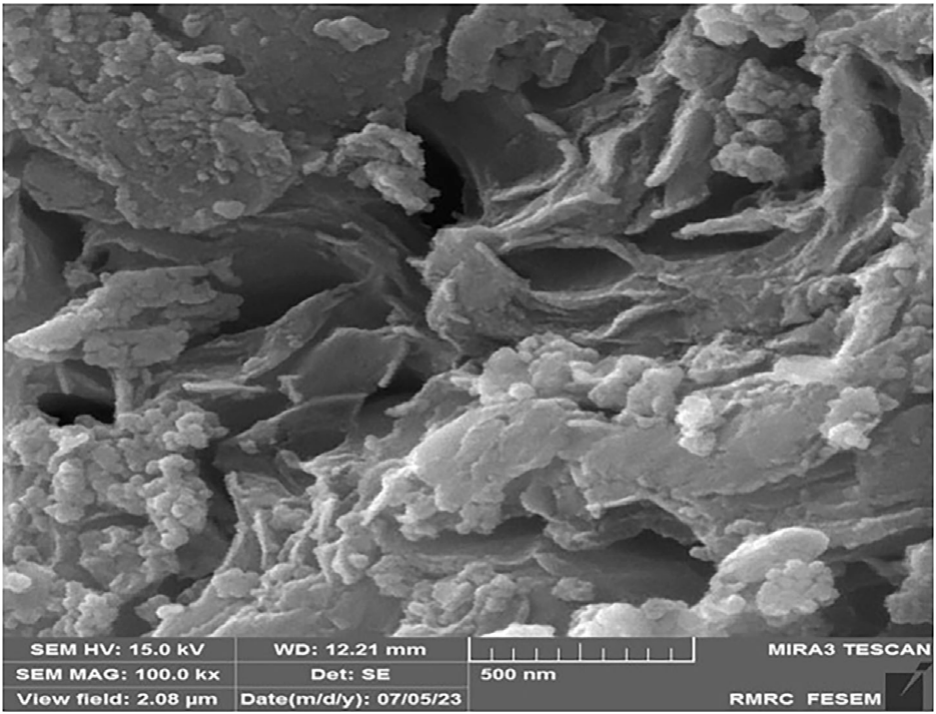
FESEM image of CoFe2O4@ZnO@Bentonite nanocatalyst.

The surface morphology of CoFe_2_O_4_@ZnO@Bentonite nanostructures was evaluated by scanning electron microscope (SEM) and related images are presented in Figure 2. Accordingly, CoFe_2_O_4_@ZnO@Bentonite nanocatalysts presented a two-dimensional plate-shaped morphology with almost uniform size distribution.

**FIGURE 3:**
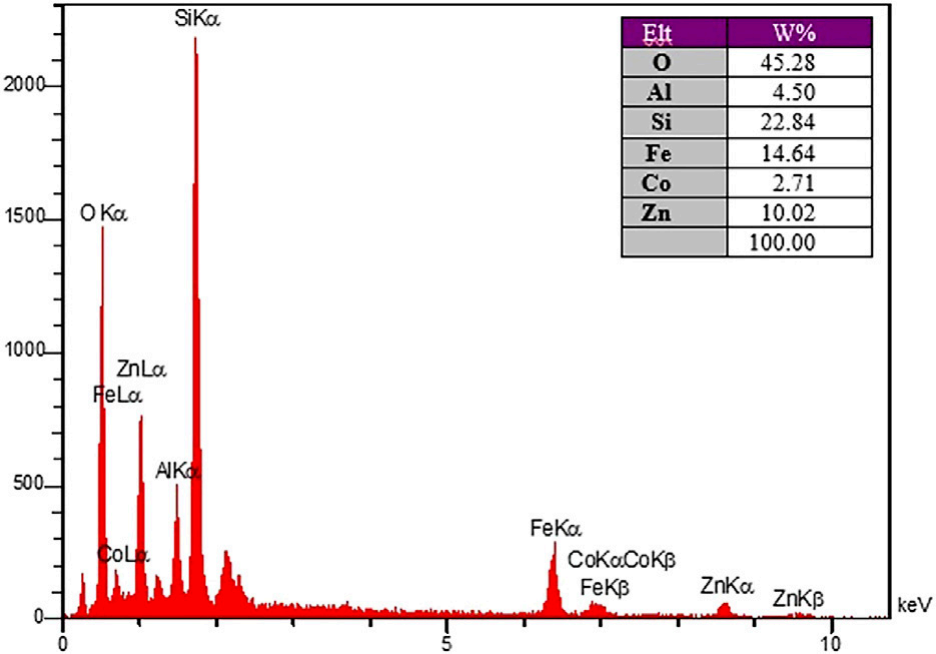
EDX analysis spectrum to determine the constituent elements of CoFe2O4@ZnO@Bentonite nanocatalyst.

The purity of CoFe_2_O_4_@ZnO@Bentonite nanocomposite was analyzed through X-ray energy diffraction (EDX) elemental analysis (Figure 3). The results showed the distribution of C, N, O, Co, Fe, and Zn in the composition of the final nanocatalyst. Based on the results, apart from the composition of the substrate on which the tests were performed, no other impurities were observed in the structure.

**FIGURE 4:**
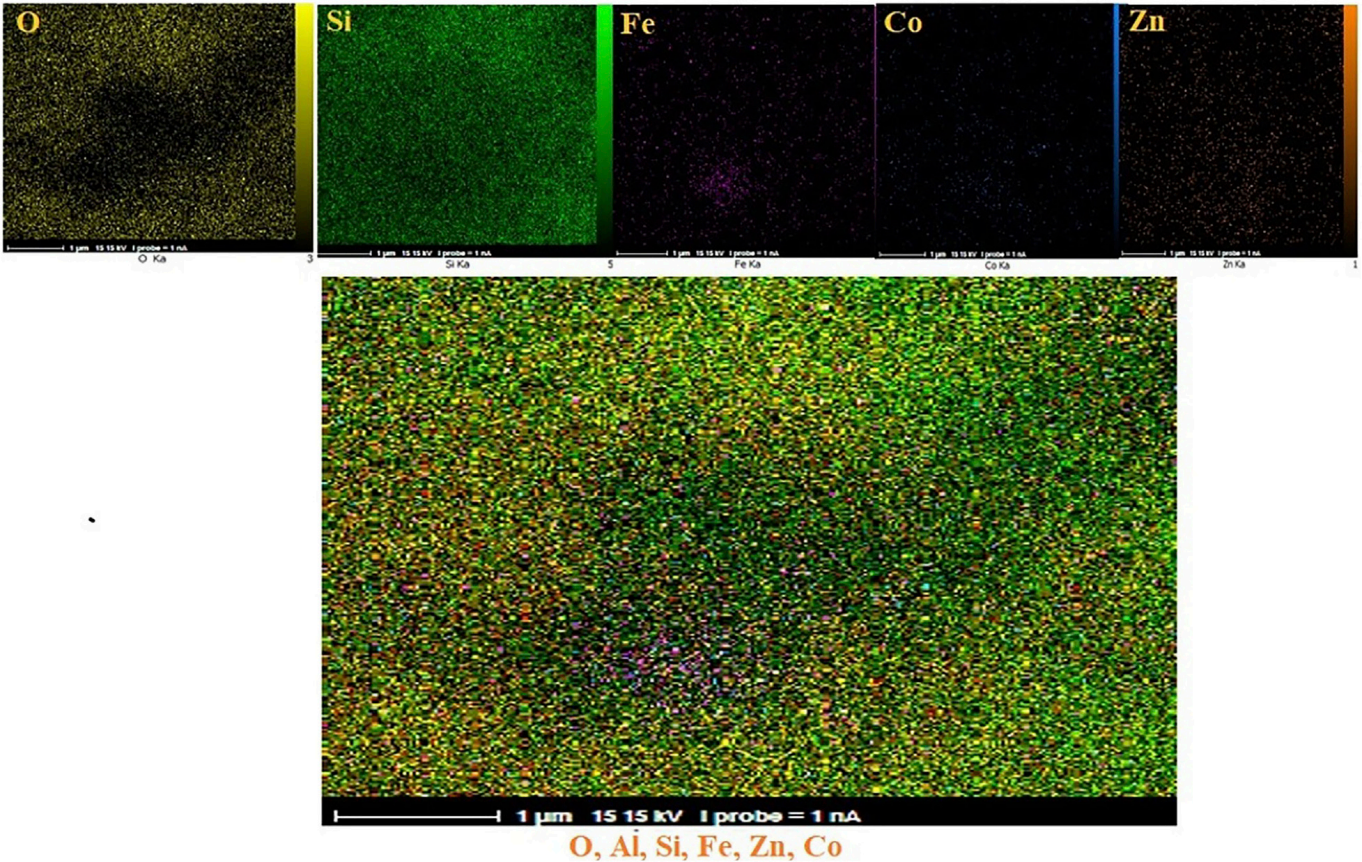
Elemental mapping of CoFe2O4@ZnO@Bentonite nanocatalyst.

The distribution of elements on the surface of CoFe_2_O_4_@ZnO@Bentonite nanostructure was examined by surface mapping analysis. Figure 4 confirms the almost homogeneous distribution of these elements on the surface of the nanocatalyst.

**FIGURE 5:**
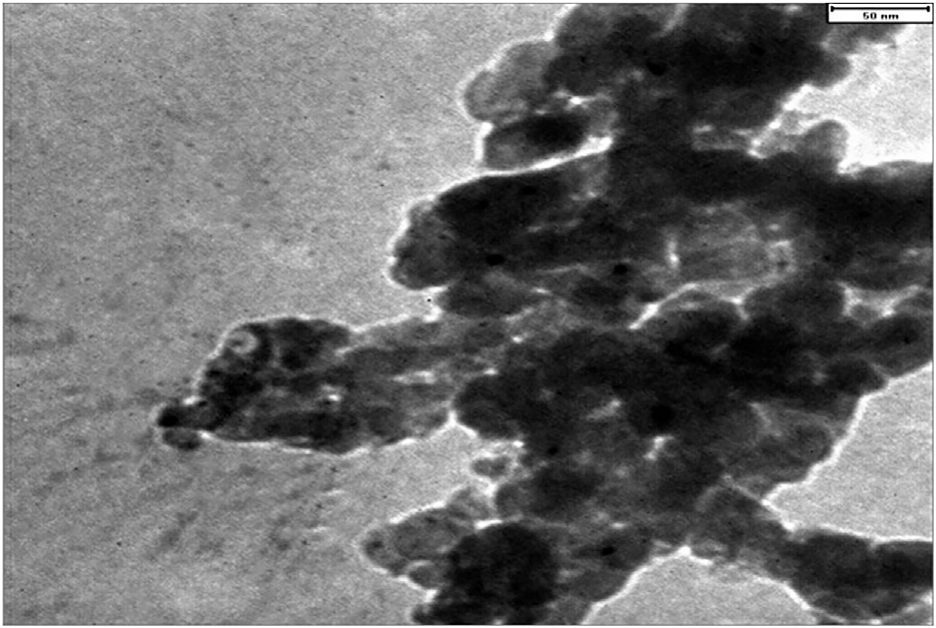
Transmission electron microscope image to investigate the morphology and particle size distribution in CoFe2O4@ZnO@Bentonite nanocatalyst.

TEM was employed to investigate the particle size distribution and the stability of CoFe_2_O_4_@ZnO@Bentonite nanostructure in particle adhesion. As shown in Figure 5, the constituent particles of this nanocatalyst have nanoscale size. This figure also shows the formation of two-dimensional sheets in CoFe_2_O_4_@ZnO@Bentonite nanocatalyst, which is in agreement with the SEM results. No agglomeration of particles, suggesting the high stability of the nanoparticles forming the CoFe_2_O_4_@ZnO@Bentonite catalyst. The synthesis of samples with uniform morphology, narrow size distribution, and high-stability surfaces facilitates their application in various fields including catalytic applications.

**FIGURE 6:**
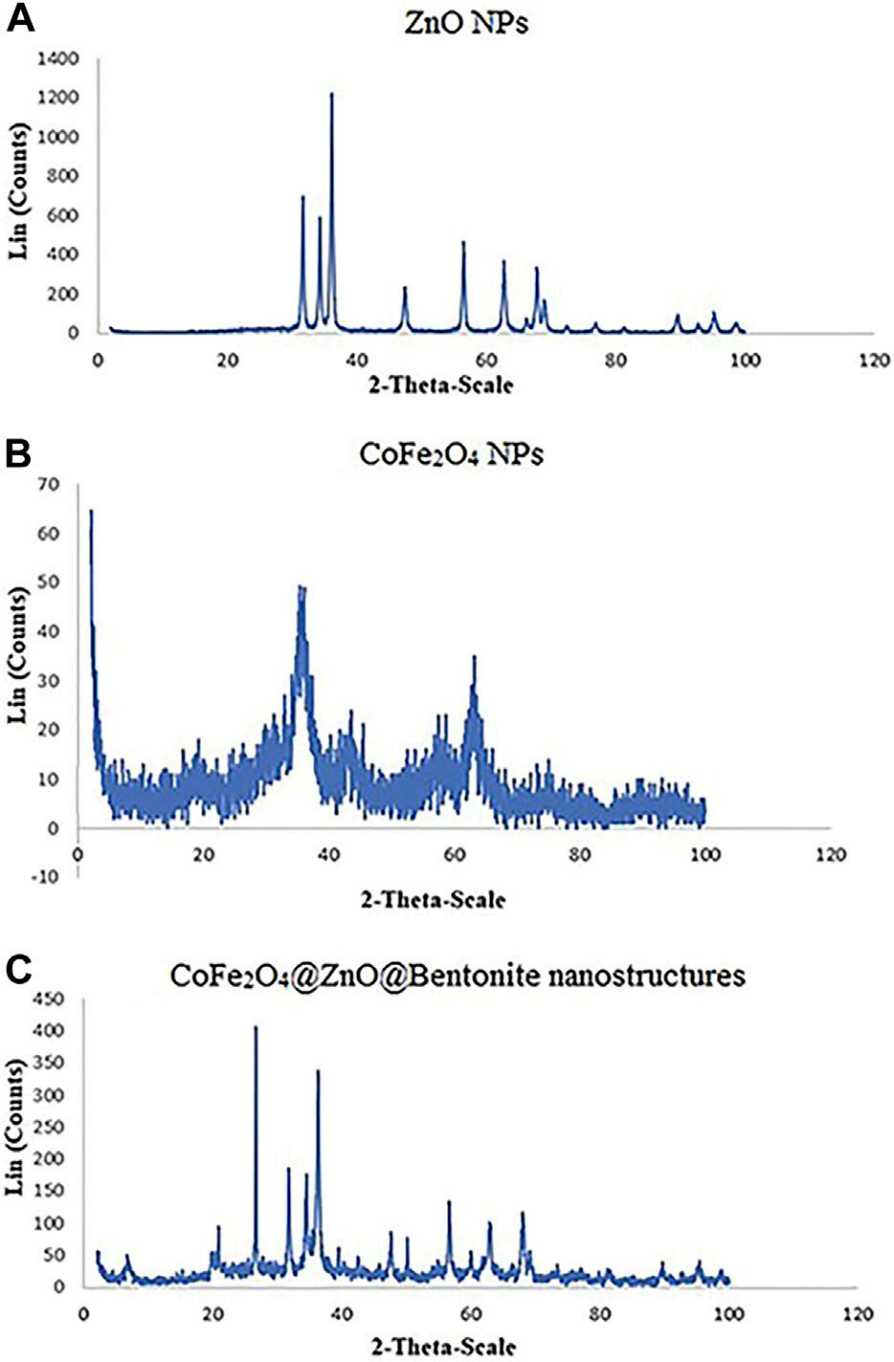
X-ray diffraction pattern (XRD) **(A)** ZnO nanoparticle, **(B)** CoFe2O4 nanoparticle, and **(C)** CoFe2O4@ZnO@Bentonite nanostructures.

Figures 6A–C show the XRD patterns of ZnO NPs, CoFe_2_O_4_ NPs, and CoFe_2_O_4_@ZnO@Bentonite nanocatalyst. Based on the results, the characteristic peaks related to the formation of the ZnO nanostructure are in agreement with previous studies, confirming the presence of the diffraction peaks of this nanostructure (Gerbreders et al., 2020; Shkir et al., 2019). The diffraction pattern of CoFe_2_O_4_ nanostructures coincides with previous studies, providing strong evidence to confirm these nanostructures (Al-Gethami et al., 2022; Revathi et al., 2020). The XRD pattern of the CoFe_2_O_4_@ZnO@Bentonite nanocatalyst (Figure. 6C) shows the characteristic peaks of both ZnO and CoFe_2_O_4_, confirming the correct synthesis of the CoFe_2_O_4_@ZnO@Bentonite nanocatalyst. The absence of an additional peak in the diffraction pattern of the nanocatalyst also indicates the high purity of the samples, therefore, ZnO and CoFe_2_O_4_ are actively present in the final structure of the catalyst with no specific chemical reaction. Moreover, based on the Debye-Scherer equation, the broad peaks in the final structure of CoFe_2_O_4_@ZnO@Bentonite indicate its narrow size distribution. As crystals are the basis in the formation of particles, the synthetic crystals in this study provide the basis for the formation of nanocrystalline sheets of the CoFe_2_O_4_@ZnO@Bentonite nanocatalyst, which in complete accordance with the SEM results.

**FIGURE 7:**
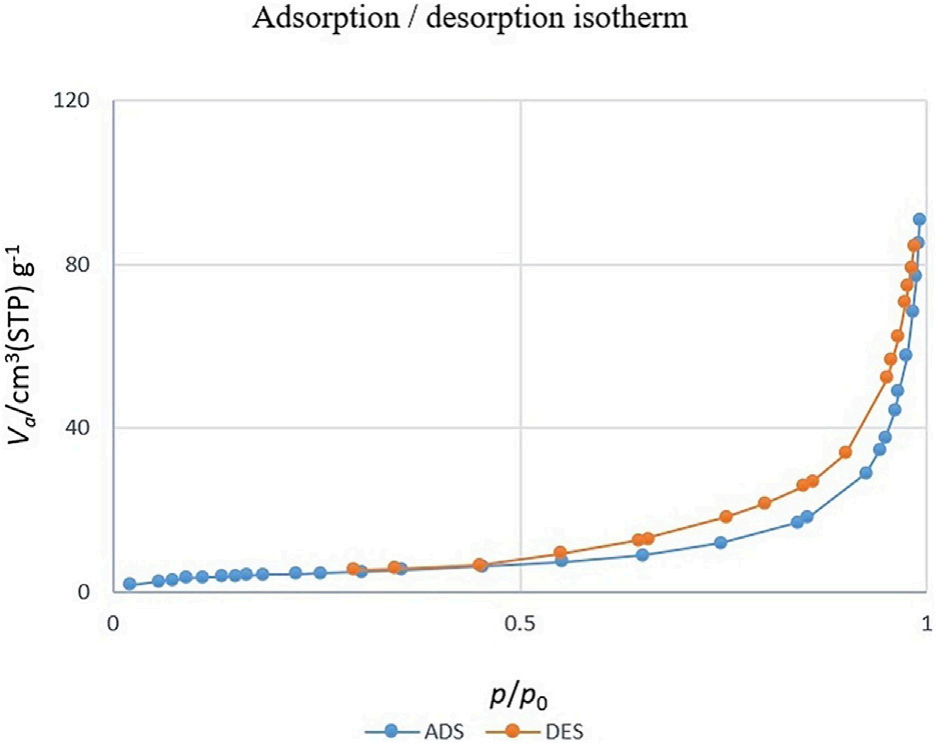
Nitrogen adsorption and desorption isotherm related to CoFe2O4@ZnO@Bentonite nanocatalyst.

Figure 7 shows the nitrogen adsorption-desorption isotherm of CoFe_2_O_4_@ZnO@Bentonite nanocatalysts. According to the results, the nitrogen absorption-desorption behavior of the sample is similar to the type V isotherm, suggesting the presence of micro and mesoporous structures for the final products (Sing, 2001; Medrano et al., 2022). Based on the BET technique, the surface area of CoFe_2_O_4_@ZnO@Bentonite nanocatalyst is 16.2 m^2^/g, confirming the optimal surface area for catalytic applications. According to Table 1, the average pore diameter of the CoFe_2_O_4_@ZnO@Bentonite sample is estimated to be 33.13 nm, confirming the mesoporous structure of the synthetic nanostructures. Moreover, the average pore volume is estimated to be 0.135 cm^3^/g, indicating the synthesis of porous catalytic samples with ideal pore volume. The synthesis of porous samples with appropriate specific surface area promotes the application of these nanostructures in the field of catalysis.

**TABLE 1:** Parameters obtained from nitrogen adsorption and desorption for nanocatalyst CoFe_2_O_4_@ZnO@Bentonite.

Surface area/(m3/g) BET	Average pore volume/(cm3/g) BJH	The average pore diameter/(nm) BJH	Sample
16.2	0.135	33.13	CoFe2O4@ZnO@Bentonite

**FIGURE 8:**
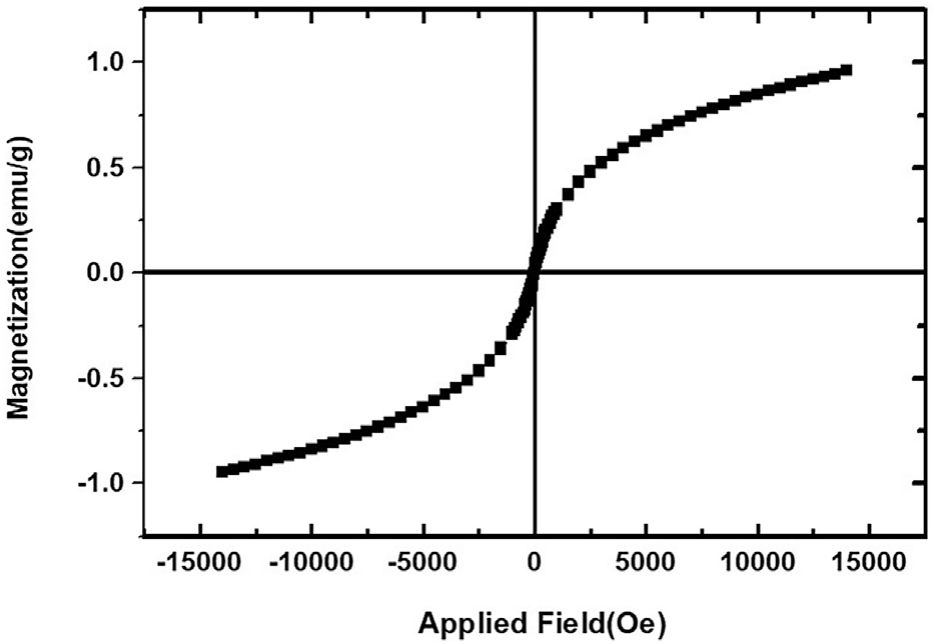
Magnetic behavior of CoFe2O4@ZnO@Bentonite nanocatalyst.

The magnetic properties of CoFe_2_O_4_@ZnO@Bentonite nanocatalysts are explored in Figure 8 shows the hysteresis of the synthesized nanocatalysts at the temperature of 25^°^C which was prepared using ZnO and CoFe_2_O_4_ nanostructures. Based on the results, CoFe_2_O_4_@ZnO@Bentonite nanocatalysts exhibited superparamagnetic properties with a saturation magnetization of 1 g/emu and an Orsted coercive force of about zero (0 Oe). The synthesis of nanostructures with desirable supermagnetic properties enhances the efficiency of samples in various fields such as catalytic processes. In addition to the type of raw materials that can affect the magnetic properties of the final products, the experimental conditions also influence the optimization and stability of these properties. Previous studies showed that the magnetic properties of the sample rose with temperature enhancement which affected the magnetic saturation and the coercive force, as well as the residual magnetization. Therefore, optimal synthesis conditions increase the performance of CoFe_2_O_4_@ZnO@Bentonite nanocatalyst.

**FIGURE 9:**
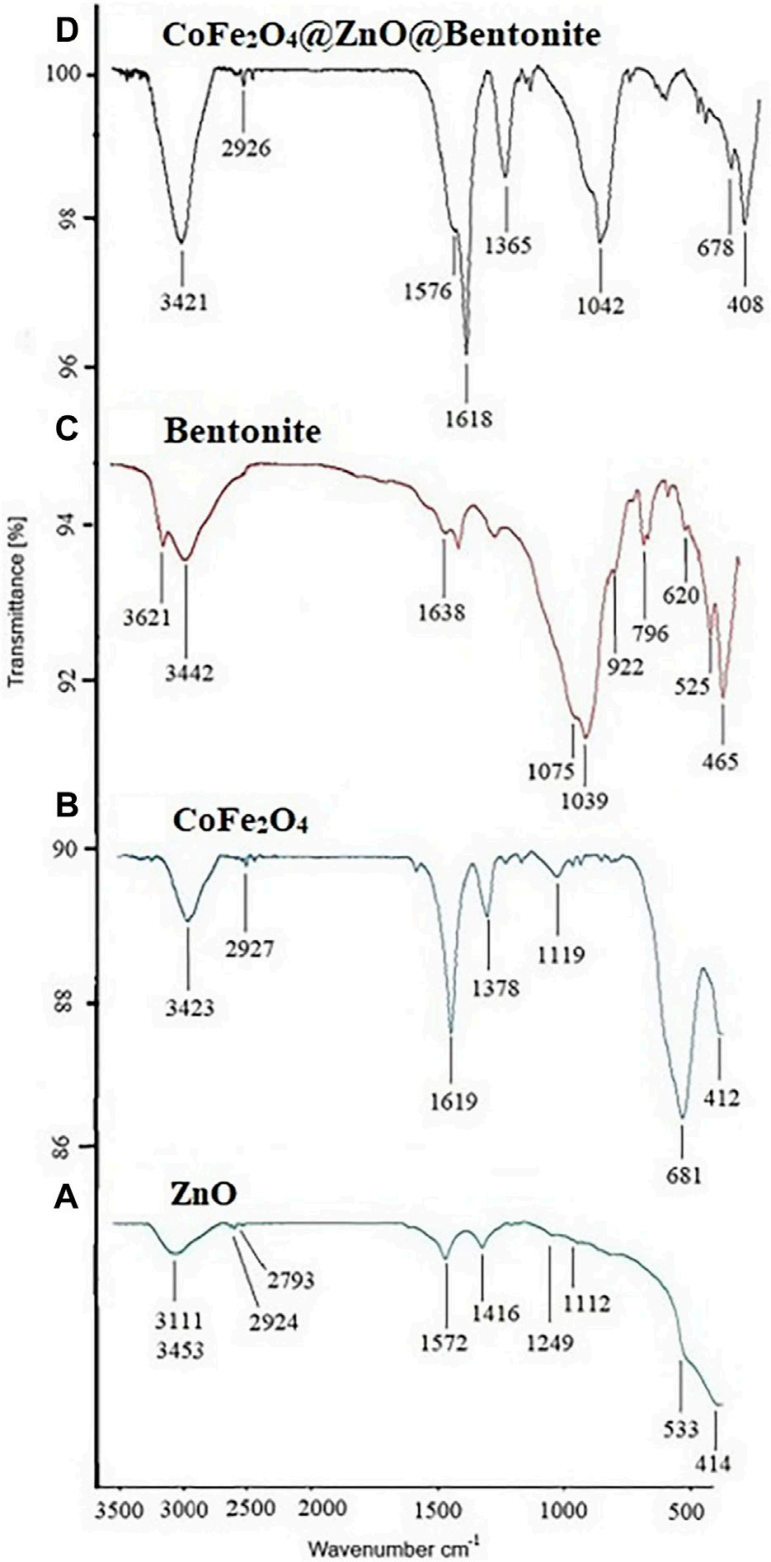
FT-IR spectrum **(A)** ZnO nanoparticle, **(B)** CoFe2O4 nanoparticle **(C)** bentonite, and **(D)** CoFe_2_O_4_@ZnO@Bentonite nanocatalyst.

FT-IR spectra of ZnO and CoFe_2_O_4_ nanoparticles and CoFe_2_O_4_@ZnO@Bentonite catalyst are depicted in Figure 9. The FT-IR spectrum of ZnO nanoparticles shows the absorption bands at 414–533 cm^−1^ corresponding to the stretching vibration of zinc oxide nanoparticles (ZnO) whereas the absorption peaks at 3,453 and 1,572 cm^−1^ are respectively attributable to the stretching and bending vibrations of hydroxyl groups adsorbed on the surface and small amounts of water adsorbed by the ZnO nanostructure (green curve) (Safaei-Ghomi et al., 2013). Moreover, the peak at 1,416 cm^−1^ is attributed to the combined bending vibrations of zinc (Zn) atoms with -OH groups (curve A).

The broad bands at 3,423 cm^−1^ are assigned to the stretching vibrations of the free or absorbed water on the surface of cobalt ferrite nanoparticles. The O–H stretching vibrations interacting through H bonds are observed at 2,927 cm^−1^ and the large absorption band present at about 1,619 cm^−1^ is due to the bending vibration of the absorbed water molecules, while the absorption band at 1,119 cm^−1^ is characteristic of the cobalt ferrite system. The absorption bands at 681 and 412 cm^−1^ can be respectively ascribed to the stretching vibrations of the metal oxide in the octahedral group Co (II)-O^2−^ and the tetrahedral group Fe (III)-O^2−^ of the cobalt ferrite complex, confirming the presence of spinel ferrite (curve B) (Boobalan et al., 2013; Rana et al., 2010; Cannas et al., 2010).

The absorptions appearing in the FT-IR spectrum of bentonite are: Si-O-Si bending at 468 cm^−1^, tetrahedral SiO_4_ bending and Al-O-Si bending at 525 cm^−1^, Si-O and Al-O out-of-plane bending at 620 cm^−1^, tetrahedral SiO_4_ bending at 796 cm^−1^, deformation of Al-Al-OH or Al-OH-Al species at 922 cm^−1^, tetrahedral SiO_4_ stretching at 1,039 cm^−1^ and 1,075 cm^−1^, water H-O-H bending appeared at 1,638 cm^−1^. The broad peak at 3,442 cm^−1^ along with a single peak at 3,621 cm^−1^ respectively belongs to the O-H frequency of the silanol groups Si-O-H and the stretching O-H of the hydroxyl groups of the structure and water in the mineral substance, and this indicates the possibility of bonding Hydroxyl is between octahedral and tetrahedral layers (curve C) (Zaitan et al., 2008; Ravindra Reddy et al., 2017).

In the FT-IR spectrum of the final CoFe_2_O_4_@ZnO@Bentonite nanocatalyst, all the characteristic peaks of two ZnO and CoFe_2_O_4_ nanoparticles can be seen. In the nanocomposite, absorption 408 cm^−1^ indicates the presence of Zn-O or CoFe_2_O_4_ nanoparticles, absorption 678, 1,618, and 1,365 cm^−1^ indicate the presence of cobalt ferrite and absorption 1,042 cm^-1^ indicates cobalt ferrite or ZnO. The peaks observed in the regions of 3,421 and 1,576 cm^−1^ are related to the vibrations of the hydroxy group (OH) of moisture absorbed on the surface of the nanocomposite (curve D). The absorption bands at 620, 796, 922, and 1,039 cm^−1^ respectively correspond to Al-O and Si-O out-of-plane bending, tetrahedral SiO_4_ bending, deformation of Al-Al-OH species or OH-Al-AL and tensile SiO_4_ are tetrahedral in bentonite bed (curve C). Minor changes in the location of these adsorptions indicate their interaction with bentonite.

### 3.2 Preparation of tetrahydrobenzo(a)xanthan derivatives using Ag_2_O NP@IOP nanocatalyst

**SCHEME 2:**
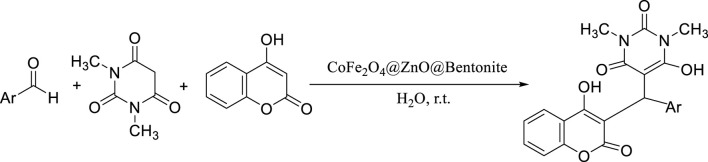
Synthesis of benzylbarbiturocoumarins in the presence of CoFe_2_O_4_@ZnO@Bentonite nanocatalyst.

Among the different kinds of catalysis, heterogeneous catalysts have more importance in the industrial production of valuable products for various reasons such as their longevity, ease of separation, and reusability. After designing, synthesizing, and identifying CoFe_2_O_4_@ZnO@Bentonite nanocomposite, its efficiency as a catalyst was investigated in preparation of benzylbarbiturocoumarins by one-step and three-component reaction of 4-hydroxycoumarin, 1,3-dimethylbarbituric acid, and different aromatic aldehydes (Scheme 2).

To determine the optimal and suitable conditions for the synthesis of benzylbarbiturocoumarins, first, the reaction conditions were investigated in terms of a suitable solvent, temperature, and nanocatalyst amount. For this purpose, the model reaction of 3-nitrobenzaldehyde (3 mmol), 1,3-dimethylbarbituric acid (3 mmol), and 4-hydroxycoumarin (3 mmol) was selected in the presence of 15 wt.% CoFe_2_O_4_@ZnO@Bentonite nanocatalyst and reflux conditions.

To investigate the effect of the solvent, the model reaction was carried out in the presence of various solvents such as water, ethanol, methanol, water: ethanol (1:1), methanol: water (1:1), dimethylformamide, and acetonitrile at reflux conditions as well as solvent-free conditions at reflux conditions using 15 wt.% catalysts. The results show that the highest efficiency in the shortest time can be achieved using green water solvent (Table 2, entry 2).

**TABLE 2:** Optimization of reaction conditions^a^ for the preparation of benzylbarbiturocoumarin derivatives in the presence of CoFe_2_O_4_@ZnO@Bentonite nanocatalyst.

Entry	Catalyst	Solvent	Tem (^0^C)	Time (min/s)	Yield (%)^b^
1	CoFe_2_O_4_@ZnO@Bentonite 15 w.t.%	C_2_H_5_OH	reflux	15 min	59
2	CoFe_2_O_4_@ZnO@Bentonite 15 w.t.%	H_2_O	reflux	6 min	75
3	CoFe_2_O_4_@ZnO@Bentonite 15 w.t.%	H_2_O: C_2_H_5_OH	reflux	25 min	36
4	CoFe_2_O_4_@ZnO@Bentonite 15 w.t.%	CH_3_OH	reflux	140 min	Trace
5	CoFe_2_O_4_@ZnO@Bentonite 15 w.t.%	H_2_O: CH_3_OH	reflux	8 min	64
6	CoFe_2_O_4_@ZnO@Bentonite 15 w.t.%	CH_3_CN	reflux	10 min	57
7	CoFe_2_O_4_@ZnO@Bentonite 15 w.t.%	DMF	reflux	2 min	39
8	CoFe_2_O_4_@ZnO@Bentonite 15 w.t.%	Free-Solvent	reflux	20 min	68
9	Catalyst-free	H_2_O	reflux	24 h	N.R.
10	CoFe_2_O_4_@ZnO@Bentonite 10 w.t.%	H_2_O	reflux	10 min	66
11	CoFe_2_O_4_@ZnO@Bentonite 20 w.t.%	H_2_O	reflux	2 min	97
12	CoFe_2_O_4_@ZnO@Bentonite 25 w.t.%	H_2_O	reflux	2 min	95
13	CoFe_2_O_4_@ZnO@Bentonite 20 w.t.%	H_2_O	r.t.	1 min	97
14	CoFe_2_O_4_@ZnO@Bentonite 20 w.t.%	H_2_O	40	1 min	97
15	CoFe_2_O_4_@ZnO@Bentonite 20 w.t.%	H_2_O	80	1 min	97
16	CoFe_2_O_4_@ZnO@Bentonite 20 w.t.%	H_2_O	r.t., UV	30 s	99

^a^ Reaction conditions: 3-nitrobenzaldehyde (4 mmol), 1,3-dimethylbarbituric acid (4 mmol), and 4-hydroxycoumarin (4 mmol) in presence of CoFe2O4@ZnO@Bentonite 20 w.t.% under different conditions.

^b^ Yield refer to isolated products.

To optimize the catalyst amount to achieve maximum efficiency at the minimum amount of catalyst, the model reaction was evaluated in the absence of a catalyst and the presence of different amounts of CoFe_2_O_4_@ZnO@Bentonite nanocatalyst. According to Table 2, the presence of a catalyst was necessary to proceed with the reaction, such that, the reaction did not take place without the catalyst even after 24 h (Table 2, entry 9). Moreover, 20 wt.% CoFe_2_O_4_@ZnO@Bentonite nanocatalyst was chosen as the optimal content for the synthesis of benzylbarbiturocoumarin derivatives (Table 2, entry 11).

After determining the optimal solvent and catalyst content, the effect of temperature was assessed on this model reaction using 15 mL of distilled water as a solvent and 20 wt.% CoFe_2_O_4_@ZnO@Bentonite nanocatalyst at ambient temperature, 40, 80°C, and reflux conditions. Based on Table 2 (entry 13), the ambient temperature was selected as the optimal temperature. Also, the reaction was carried out in the presence of ultraviolet light. UV light have many advantages and the progress of the reaction improved in the presence of ultraviolet light, but since excessive use and long-term contact with ultraviolet radiation causes damage to the body, especially the skin and eyes, and increases the risk of skin problems such as malignant melanoma and eye injuries such as pterygium, cataracts, inflammation and corneal irritation., we preferred not to consider the optimal reaction conditions with ultraviolet light and not to change the optimal conditions (ambient temperature, water solvent and 20% w.t. catalyst).

To determine the optimal reaction conditions and to confirm the generalizability of this protocol, various benzylbarbiturocoumarin derivatives were prepared using aromatic aldehydes with different substituent groups and electron density, 1,3-dimethylbarbituric acid, and 4-hydroxycoumarin. The time and efficiency of the reactions are listed in Table 3. The results showed that aldehydes with different substituent groups and electron density and 1,3-dimethylbarbituric acids can lead to the production of the corresponding products at high yields. All the prepared compounds were characterized by melting point and IR spectroscopy, and compound 4d was checked by ^1^H NMR and ^13^C NMR spectroscopy for further certainty.

**TABLE 3:** Preparation of benzylbarbiturocoumarin derivatives in the presence of CoFe2O4@ZnO@Bentonite nanocatalyst.

Entry	R (aldehyde)	Rʺ	Product	Time (min)	Yield (%)	$\frac{m.p.\;\left(℃\right)}{Observed\;Reported\;\left[ref.\right]}$	
1	C_6_H_5_-	CH_3_	4a	3	94	190–194	189–190 (Gerbreders et al., 2020)
2	4-NO_2_C_6_H_4_-	CH_3_	4b	2	92	193–194	193–194 (Gerbreders et al., 2020)
3	2-ClC_6_H_4_-	CH_3_	4c	5	90	195–196	198–200 (Gerbreders et al., 2020)
4	3-NO_2_C_6_H_4_-	CH_3_	4d	1	97	193–194	195–196 (Gerbreders et al., 2020)
5	4-OCH_3_C_6_H_4_-	CH_3_	4e	1	78	186–188	186–188 (Gerbreders et al., 2020)
6	4-CH_3_C_6_H_4_-	CH_3_	4f	3	93	195–198	193–194 (Gerbreders et al., 2020)
7	5-Br-2-OHC_6_H_3_-	CH_3_	4g	4	97	190–192	New

^a^ Reaction conditions: Aryl aldehyde (4 mmol), 1,3-dimethylbarbituric acid (4 mmol), and 4-hydroxycoumarin (4 mmol) in the presence of CoFe2O4@ZnO@Bentonite (20 w.t.%) in H2O at temperature of 40°C.

^b^ Isolated yields after purification.

**SCHEME 3:**
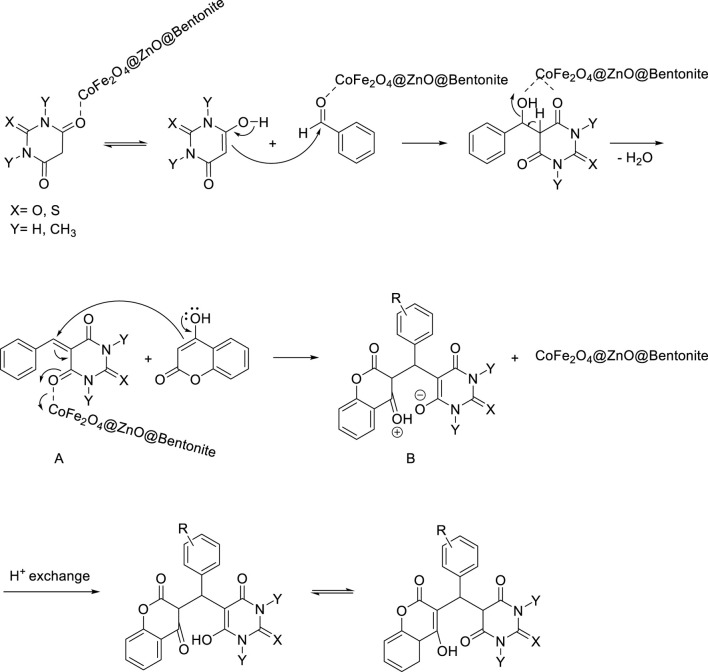
Mechanism of formation of benzylbarbiturocoumarins.

The proposed reaction mechanism is shown in Scheme 3. Lewis acid nanocatalyst of CoFe_2_O_4_@ZnO@Bentonite increases the electrophilic character of carbonyl aldehyde and barbituric acid groups through a strong coordination bond causing the oxygen atom of aldehyde in the vicinity of this active catalyst and the carbon of carbonyl aldehyde to become a good electrophilic center. On the other hand, the oxygen atom of barbituric acid turns into the corresponding enol form. In the next step, the enolic form of barbituric acid attacks the carbon atom of the activated aldehyde group through a carbon atom and produces intermediate α, *β*-unsaturated arylidene barbituric acid (A) by Knoevenagel condensation reaction. Then, 4-hydroxycoumarin is added to the activated condensation intermediate in the presence of CoFe_2_O_4_@ZnO@Bentonite Lewis acid nanocatalyst during a Michael addition reaction to form intermediate B, which finally forms the final product through a proton exchange step and keto-enol tautomerism.

**FIGURE 10:**
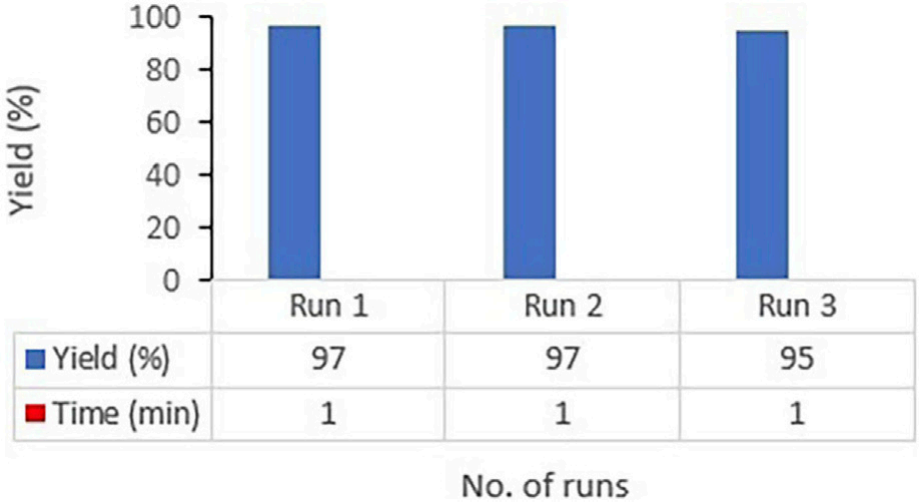
Recovery of CoFe2O4@ZnO@Bentonite nanocatalyst in the preparation of compound 4d.

To assess the recyclability of synthesized catalyst, the model reaction of 3-nitrobenzaldehyde, 1,3-dimethylbarbituric acid, and 4-hydroxycoumarin was performed for the synthesis of 6-hydroxy-5-(2-(4-hydroxy-2-oxo-2H-chromen-3-yl)-1-(3-nitrophenyl)ethyl)-1,3-dimethylpyrimidine-2,4(1H,3H)-dione (4d)) once in the presence of fresh CoFe_2_O_4_@ZnO@Bentonite catalyst. By tracking the completion of the reaction time by TLC, at the end of the reaction and after filtering the reaction mixture, the precipitates on the filter were dissolved in dimethyl sulfoxide (DMSO) solvent. As the product is dissolved in DMSO solvent, but the catalyst is not, the catalyst can be easily separated through a simple filtration and after washing with ethanol and drying, the catalyst was used for the next round. The results of recycling the catalyst for three consecutive cycles showed that the catalyst can be recovered with a small reduction in its catalytic activity (Figure 10).

#### 3.2.1 Characterization of CoFe_2_O_4_@ZnO@Bentonite nanocatalyst after recovery process

**FIGURE 11:**
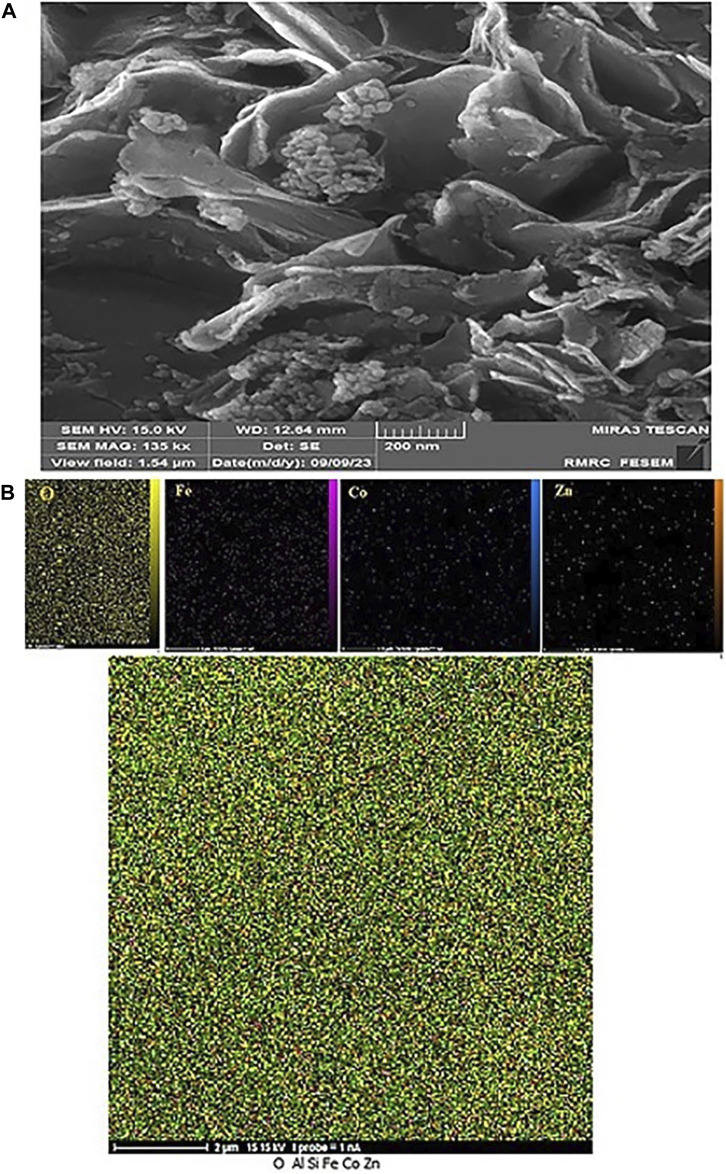
Image **(A)** Scanning electron microscope and **(B)** Surface mapping of CoFe2O4@ZnO@Bentonite nanocatalyst after the recovery process.

**FIGURE 12:**
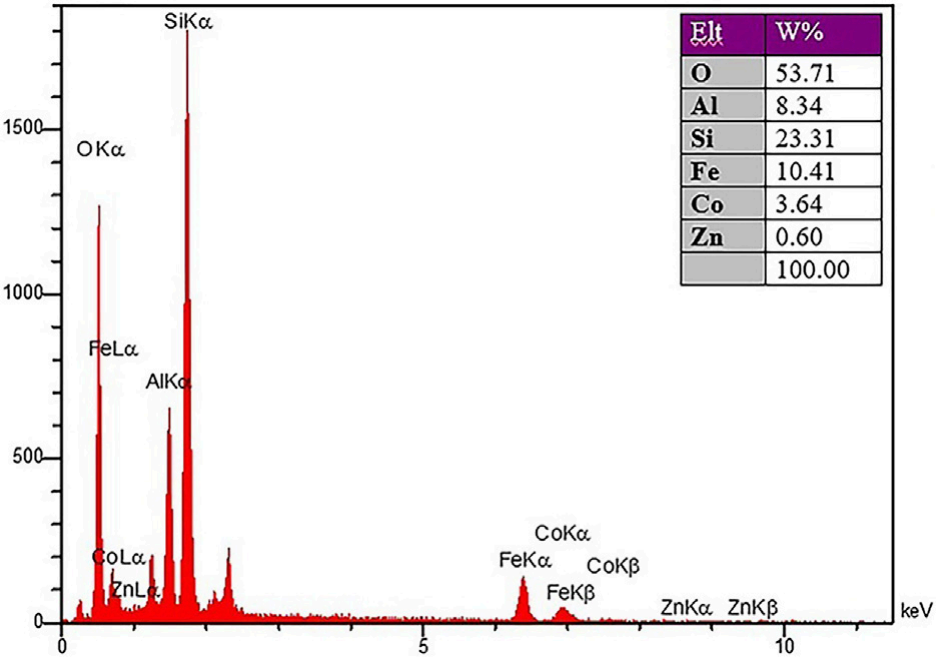
X-ray energy diffraction analysis spectrum of CoFe_2_O_4_@ZnO@Bentonite nanocatalyst after the recovery process.

To check the morphology, surface properties, and stability of the sample after the catalyst recovery, scanning electron microscopy (SEM), surface mapping analysis, and EDX elemental analysis were employed. Based on the SEM results, the sample has a nanocrystalline plate morphology, which is in agreement with the morphology of the CoFe_2_O_4_@ZnO@Bentonite nanocatalyst before the recovery process (Figure 11A). This image shows evidence of aggregation of the structure on the surface of the sample which is related to the performance of the nanocatalyst during the catalytic process. As known, this instability is completely temporary and related to the van der Waals reactions of the nanocatalyst during the production of benzyl-barbiturocoumarin derivatives. Mapping analysis of the surface also indicates the presence of the constituent elements of the nanocatalyst in the final structure, suggesting the stability of the nanocatalyst structure after the recovery (Figure 11B). This analysis confirms that no specific side reaction occurred on the surface of the compound after the recovery process which is by the original sample and before the recovery process. EDX elemental analysis was carried out to quantitatively evaluate the constituent elements of the nanocatalyst after the recovery process (Figure 12) and the content of each constituent element. As an important result, the quantity of the constituent elements of the nanocatalyst after the recovery process is almost the same as their contents before the recovery process, which confirms the optimal performance of the nanostructure in the catalytic process.

**FIGURE 13:**
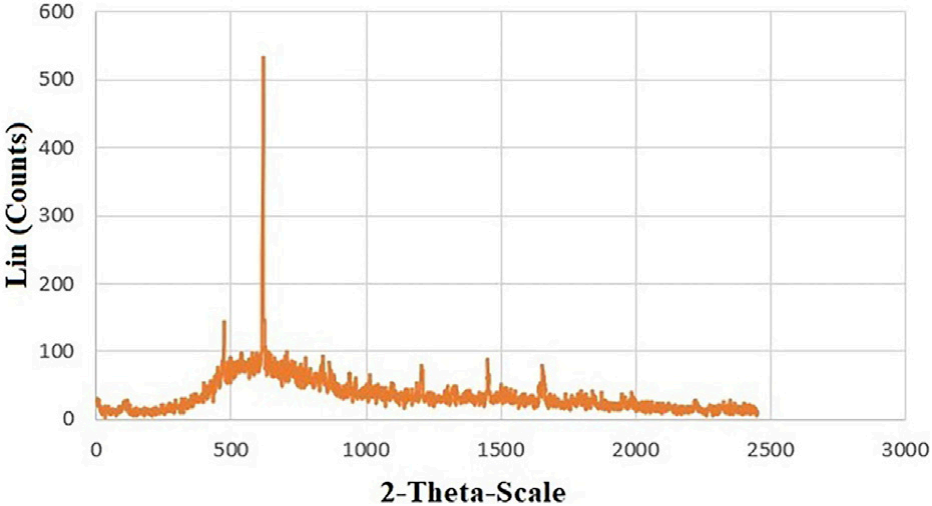
XRD pattern of CoFe_2_O_4_@ZnO@Bentonite nanocatalyst after the catalytic recovery process.

The XRD patterns of CoFe_2_O_4_@ZnO@Bentonite nanocatalyst are depicted in Figure 13. Based on the results, the characteristic peaks related to the formation of CoFe_2_O_4_@ZnO@Bentonite crystals coincide with the sample before the catalyst reduction process. The absence of an extra peak in the diffraction pattern of CoFe_2_O_4_@ZnO@Bentonite nanocatalyst indicates the high performance of the product after recovery process. It also indicates that the ZnO and CoFe_2_O_4_ nanostructures had no specific side chemical reaction in the final structure of CoFe_2_O_4_@ZnO@Bentonite. Based on the Debye-Scherer equation, the distribution of peaks in the structure of the recovered nanocatalyst was sharper than the sample before recovery, suggesting the effect of the catalytic performance CoFe_2_O_4_@ZnO@Bentonite on the size distribution of the peaks.

Some of the previously reported protocols in this field are listed in Table 4. To compare the time required to perform the reaction, the use of organic solvents, the reaction conditions and the yield are presented for preparing 6-hydroxy-5-(2-(4-hydroxy-2-oxo-H2-chromen-3-yl)-1-(3-nitrophenyl)ethyl)-1,3-dimethylpyrimidine- 2,4(1H,3H)-dione (4d). The limitations in some of these methods, such as long reaction time and reflux temperature, motivated us to design and present a new approach to preparing benzylbarbiturocoumarins. Based on the results, the proposed method is more favorable in terms of short reaction time and higher efficiency. This method is environmentally friendly and following green chemistry due to the use of natural bentonite substrate and the preparation of benzylbarbiturocoumarins in water solvent.

**TABLE 4:** Comparison of the efficiency of CoFe2O4@ZnO@Bentonite nanocatalyst with several catalysts and previous methods presented in the preparation of benzylbarbiturocoumarin derivatives.

Entry	Catalyst	Amount of catalyst	Conditions	Time (min/h)	Yield (%)	Ref
1	SSC	1 mol%	H_2_O, 80°C	130	82	(Gerbreders et al., 2020)
2	GO NSs	0.005 g	EtOH: H_2_O, 80°C	150	86	(Nabiyouni et al., 2014)
3	CoFe_2_O_4_@ZnO@Bentonite	20 w.t.%	H_2_O, r.t.	1	97	This work

## 4 Conclusion

In conclusion, the synthesis of a new heterogeneous CoFe_2_O_4_@ZnO@Bentonite nanocatalyst was successfully carried out and its characteristics were examined by FE-SEM, EDX, Mapping, XRD, BET, and VSM. It was explored in the three-component reaction of 4-hydroxycoumarin, 1,3-dimethylbarbituric acid, and aromatic aldehydes which confirmed its efficiency as a good alternative to toxic and expensive catalysts. The natural bentonite substrate, the recyclability of the nanocatalyst, and the compatibility of the reaction environment with green chemistry are the key features of this approach for the preparation of benzylbarbiturocoumarin derivatives.

## Data Availability

The original contributions presented in the study are included in the article/Supplementary Material, further inquiries can be directed to the corresponding author.
