# Supplementary material for: Synthesis of cobalt- ferrite and zinc oxide metal nanoparticles based-bentonite using SDS and their investigation as catalysts in synthesis of benzylbarbiturocoumarins
Source: Front Chem. 2024 Aug 12;12:1434488. doi: 10.3389/fchem.2024.1434488 (PMC11345271; doi:10.3389/fchem.2024.1434488)
Supplement: Supplementary file 1 [file DataSheet1.docx]

***Supplementary Material***

**Synthesis of cobalt-** **ferrite and zinc oxide metal nanoparticles based-bentonite using SDS and their investigation as catalysts in synthesis of** **benzylbarbiturocoumarins**

Pegah Baminejad^1^ . Enayatollah Sheikhhosseini^1^ . Mahdieh Yahyazadehfar^1^

E-mail: [sheikhhosseiny@gmail.com](mailto:sheikhhosseiny@gmail.com) or sheikhhosseini@iauk.ac.ir

**IR, ^1^H NMR and ^13^C NMR of compound (4g)**

5-(1-(5-bromo-2-hydroxyphenyl)-2-(4-hydroxy-2-oxo-2H-chromen-3-yl)ethyl)-6-hydroxy-1,3-dimethylpyrimidine-2,4(1H,3H)-dione (4g): Yield: 97%. M.p. = 190-192 ^°^C. IR (KBr,cm^-1^): 3473, 3365, 2967, 2933, 1675, 1652, 1603, 1568, 1425, 1385, 818, 757. ^1^H NMR (400 MHz, DMSO-*d_6_,* ppm): δ = 3.14 (s, 3H, CH_3_), 3.31 (s, 3H, CH_3_), 5.85 (s, 1H, CH), 6.67 (d, *J*_1_ = 8.4 Hz, 1H, H-Ar), 7.13 (dd, *J*_1_ = 8.8 Hz, *J*_2_ = 2 Hz 1H, H-Ar), 7.17 (s, 1H, H-Ar), 7.32 (d, *J*_1_ = 6.8 Hz, 1H, H-Ar), 7.66 (d, *J* = 8.4 Hz, 1H, H-Ar), 7.74 (t, *J* = 8.8 Hz, 2H, H-Ar) ppm, 11.66 (s, 1H, OH), 12.38 (brs, 2H, OH); ^13^C NMR (100 Hz, DMSO-*d*_6_, ppm): δ = 28.5, 30.3, 39.4, 58.4, 92.0, 104.4, 109.4, 118.0, 123.4, 124.8, 125.3, 126.3, 127.7, 128.7, 129.6, 132.6, 134.7, 152.7, 154.1, 163.5, 164.8, 168.0, 172.1.

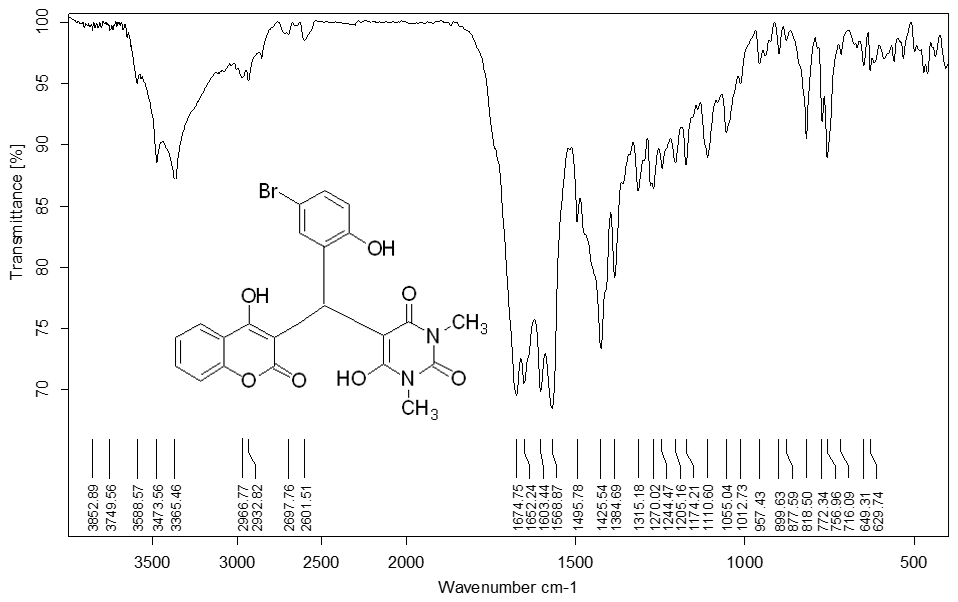


**Fig.1.** [FT-IR spectrum of compound 4g.](https://www.google.com/url?sa=t&rct=j&q=&esrc=s&source=web&cd=&cad=rja&uact=8&ved=2ahUKEwjmjuPvw579AhW_8LsIHTvtAd8QFnoECBAQAQ&url=https%3A%2F%2Fwww.researchgate.net%2Ffigure%2FThe-1-H-NMR-spectrum-of-compound-3a-in-CDCl-3-solvent_fig1_320865085&usg=AOvVaw2S68h8geITZVHy7tcJjbIy)


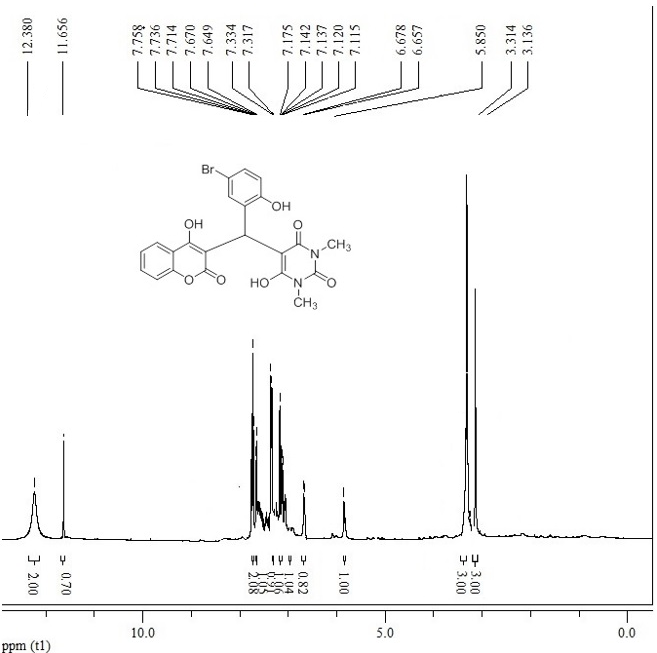


**Fig. 2.** ^[1](https://www.google.com/url?sa=t&rct=j&q=&esrc=s&source=web&cd=&cad=rja&uact=8&ved=2ahUKEwjmjuPvw579AhW_8LsIHTvtAd8QFnoECBAQAQ&url=https%3A%2F%2Fwww.researchgate.net%2Ffigure%2FThe-1-H-NMR-spectrum-of-compound-3a-in-CDCl-3-solvent_fig1_320865085&usg=AOvVaw2S68h8geITZVHy7tcJjbIy)^[H NMR spectrum of compound 4g.](https://www.google.com/url?sa=t&rct=j&q=&esrc=s&source=web&cd=&cad=rja&uact=8&ved=2ahUKEwjmjuPvw579AhW_8LsIHTvtAd8QFnoECBAQAQ&url=https%3A%2F%2Fwww.researchgate.net%2Ffigure%2FThe-1-H-NMR-spectrum-of-compound-3a-in-CDCl-3-solvent_fig1_320865085&usg=AOvVaw2S68h8geITZVHy7tcJjbIy)


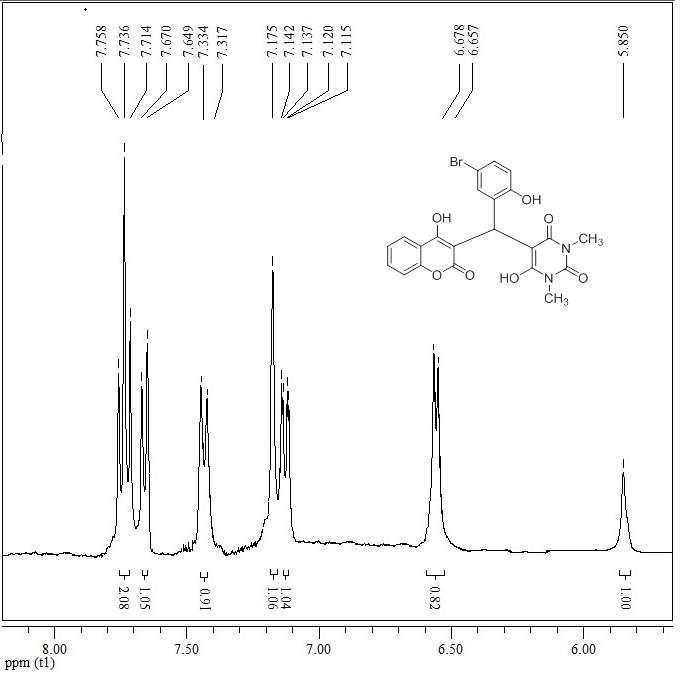


**Fig. 2a.** ^[1](https://www.google.com/url?sa=t&rct=j&q=&esrc=s&source=web&cd=&cad=rja&uact=8&ved=2ahUKEwjmjuPvw579AhW_8LsIHTvtAd8QFnoECBAQAQ&url=https%3A%2F%2Fwww.researchgate.net%2Ffigure%2FThe-1-H-NMR-spectrum-of-compound-3a-in-CDCl-3-solvent_fig1_320865085&usg=AOvVaw2S68h8geITZVHy7tcJjbIy)^[H NMR expand spectrum of compound 4g.](https://www.google.com/url?sa=t&rct=j&q=&esrc=s&source=web&cd=&cad=rja&uact=8&ved=2ahUKEwjmjuPvw579AhW_8LsIHTvtAd8QFnoECBAQAQ&url=https%3A%2F%2Fwww.researchgate.net%2Ffigure%2FThe-1-H-NMR-spectrum-of-compound-3a-in-CDCl-3-solvent_fig1_320865085&usg=AOvVaw2S68h8geITZVHy7tcJjbIy)


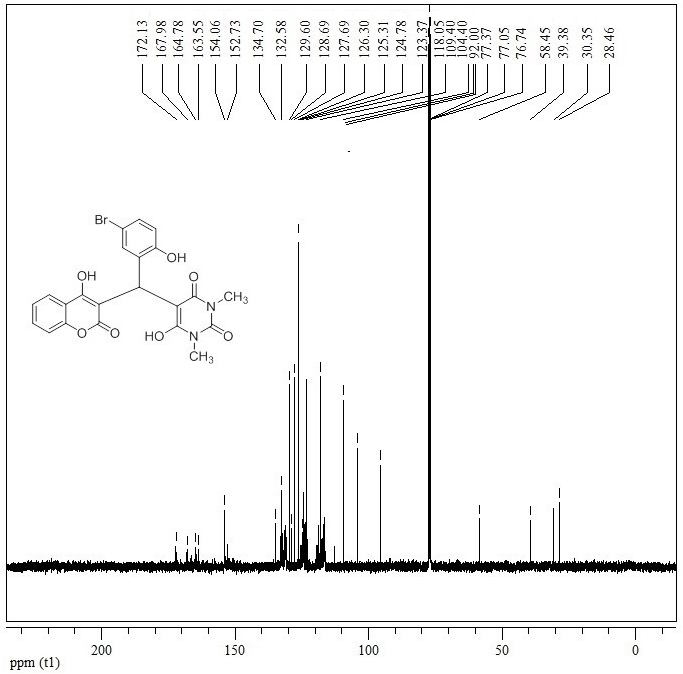


**Fig. 3.** ^[13](https://www.google.com/url?sa=t&rct=j&q=&esrc=s&source=web&cd=&cad=rja&uact=8&ved=2ahUKEwjmjuPvw579AhW_8LsIHTvtAd8QFnoECBAQAQ&url=https%3A%2F%2Fwww.researchgate.net%2Ffigure%2FThe-1-H-NMR-spectrum-of-compound-3a-in-CDCl-3-solvent_fig1_320865085&usg=AOvVaw2S68h8geITZVHy7tcJjbIy)^[C NMR spectrum of compound 4g.](https://www.google.com/url?sa=t&rct=j&q=&esrc=s&source=web&cd=&cad=rja&uact=8&ved=2ahUKEwjmjuPvw579AhW_8LsIHTvtAd8QFnoECBAQAQ&url=https%3A%2F%2Fwww.researchgate.net%2Ffigure%2FThe-1-H-NMR-spectrum-of-compound-3a-in-CDCl-3-solvent_fig1_320865085&usg=AOvVaw2S68h8geITZVHy7tcJjbIy)

**FT-IR, ^1^H NMR** **and ^13^C NMR of compound (4d)**

6-hydroxy-5-(2-(4-hydroxy-2-oxo-2H-chromen-3-yl)-1-(3-nitrophenyl)ethyl)-1,3-dimethylpyrimidine-2,4(1H,3H)-dione (4d): Yield: 95%. M.p. = 193-194 ^°^C; IR (KBr, cm^-1^): 3431, 3073, 1705, 2959, 1651, 1618, 1573, 1529, 1494, 1346, 1190, 1102, 761. ^1^H NMR (400 MHz, DMSO-*d_6_,* ppm): δ = 3.08 (s, 3H, CH_3_), 3.27 (s, 3H, CH_3_), 5.61 (s, 1H, CH), 7.54 (d, *J*_1_ = 4 Hz, 2H, H-Ar), 7.71 (d, *J*_1_ = 4 Hz, 2H, H-Ar), 7.81 (d, *J*_1_ = 8 Hz, 2H, H-Ar), 8.27 (d, *J*_1_ = 8 Hz, 1H, H-Ar), 8.73 (s, 1H, H-Ar), 12.52 (s, 1H, OH), 12.53 (s, 1H, OH). ^13^C NMR (100 Hz, DMSO-*d*_6_, ppm): δ = 28.2, 28.4, 36.2, 119.9, 120.1, 123.9, 124.0, 124.3, 129.8, 130.1, 130.3, 132.0, 132.3, 133.9, 134.0, 146.0, 146.1, 151.1, 153.6, 160.0, 161.2.

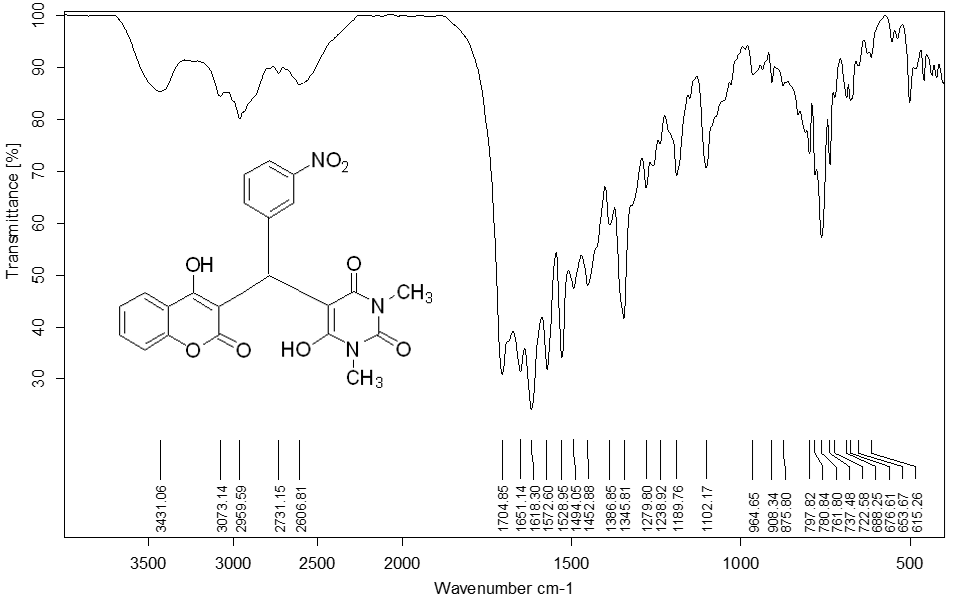


**Fig. 4.** [FT-IR spectrum of compound 4d.](https://www.google.com/url?sa=t&rct=j&q=&esrc=s&source=web&cd=&cad=rja&uact=8&ved=2ahUKEwjmjuPvw579AhW_8LsIHTvtAd8QFnoECBAQAQ&url=https%3A%2F%2Fwww.researchgate.net%2Ffigure%2FThe-1-H-NMR-spectrum-of-compound-3a-in-CDCl-3-solvent_fig1_320865085&usg=AOvVaw2S68h8geITZVHy7tcJjbIy)


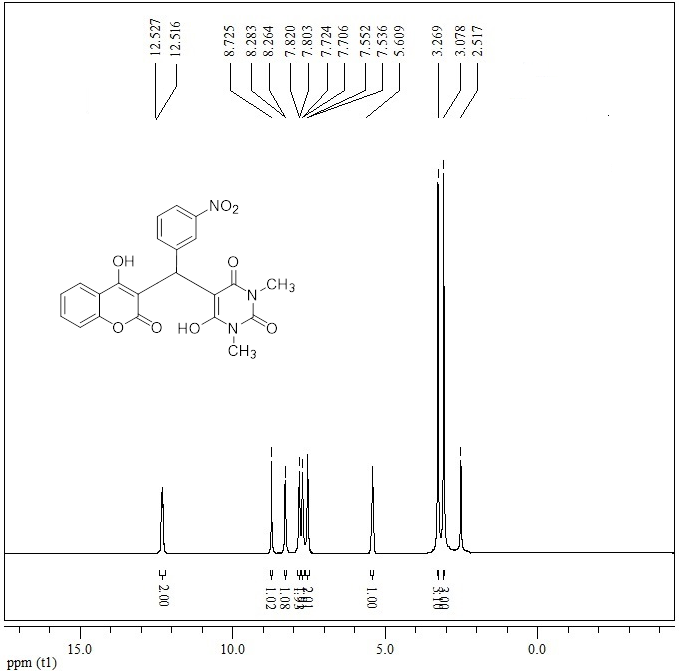


**Fig. 5.** ^1^H NMR spectrum of compound 4d.


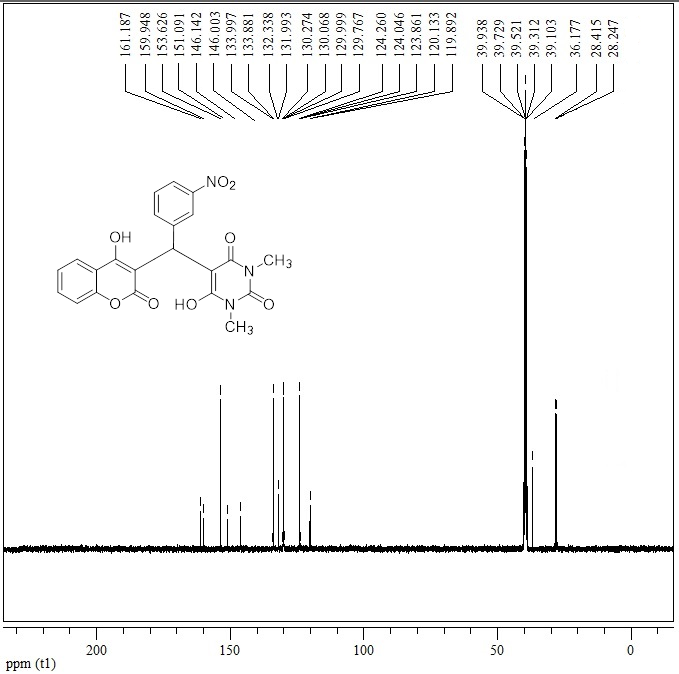


**Fig. 6.** ^13^C NMR spectrum of compound 4d.
